# Influence of High-Intensity Focused Ultrasound (HIFU) Ablation on Arteries: *Ex Vivo* Studies

**DOI:** 10.3390/mi12050485

**Published:** 2021-04-25

**Authors:** Yufeng Zhou, Wei Chun Daniel Lim

**Affiliations:** 1 School of Mechanical and Aerospace Engineering, Nanyang Technological University, Singapore 639798, Singapore; chelwc@nus.edu.sg; 2 State Key Laboratory of Ultrasound in Medicine and Engineering, College of Biomedical Engineering, Chongqing Medical University, Chongqing 400016, China; 3 Chongqing Key Laboratory of Biomedical Engineering, Chongqing Medical University, Chongqing 400016, China

**Keywords:** high-intensity focused ultrasound (HIFU), thermal lesion, mechanical effect, cavitation

## Abstract

High-intensity focused ultrasound (HIFU) has been used to ablate solid tumors and cancers. Because of the hypervascular structure of the tumor and circulating blood inside it, the interaction between the HIFU burst and vessel is a critical issue in the clinical environment. Influences on lesion production and the potential of vessel rupture were investigated in this study for the efficiency and safety of clinical ablation. An extracted porcine artery was embedded in a transparent polyacrylamide gel phantom, with bovine serum albumin (BSA) as an indicator of the thermal lesion, and degassed water was driven through the artery sample. The HIFU focus was aligned to the anterior wall, middle of the artery, and posterior wall. After HIFU ablation, the produced lesion was photographically recorded, and then its size was quantified and compared with that in the gel phantom without artery. In addition, the bubble dynamics (i.e., generation, expansion, motion, and shrinkage of bubbles and their interaction with the artery) were captured using high-speed imaging. It was found that the presence of the artery resulted in a decrease in lesion size in both the axial and lateral directions. The characteristics of the lesion are dependent on the focus alignment. Acoustic and hydrodynamic cavitation play important roles in lesion production and interaction with the artery. Both thermal and mechanical effects were found on the surface of the artery wall after HIFU ablation. However, no vessel rupture was found in this *ex vivo* study.

## 1. Introduction

Cancer is a major public health problem worldwide and the second leading cause of death. In 2020, 1,806,590 new cancer cases and 606,520 cancer deaths were predicted to occur in the United States [1]. Based on projected population aging and growth, the global burden of cancer is to increase from 18.1 million new cases in 2018 to a predicted 29.4 million cases in 2040, an increase of more than 60%, including non-melanoma skin cancerss [2]. With such a burden, there are increasing needs for better cancer treatment. High-intensity focused ultrasound (HIFU) treatment is one of the preferred treatment modalities, mostly due to its non-invasive nature and the lack of damage to the intervening tissue, especially for patients whose tumors are not suitable for surgical excision due to their difficult location [3]. Clinical applications of both benign and malignant solid tumors (i.e., breast, bone, prostate cancers, and uterine fibroids) have achieved satisfactory outcomes, and HIFU ablation has been approved in many countries, including by the Food and Drug Administration (FDA) of the United States.

Tumors usually have hypervascular structures, which means that collision of the focal region of the ultrasound with the blood vessels is highly likely. For example, it is not uncommon for liver tumors to be located adjacent to the main branches of the hepatic portal vein [4]. The circulating blood inside the arteries and blood vessels draws heat away from adjacent tissues and serves as a heat sink with great thermal diffusion for the thermal ablation, so that the temperatures drop along the outer wall of the vessel and there is a local cold spot, where the temperature and consequent thermal dose cannot reach the required value for the successful production of coagulative necrosis [5]. In addition, red blood cells are strong acoustic scatterers. Therefore, the presence of vascular structures affects the generation of an ablative lesion. Meanwhile, if a major blood vessel or artery is ruptured and the hemorrhage cannot be controlled, it is especially critical in closed procedures and may be life-threatening. Thus, this risk requires extensive investigation to better understand and improve the efficacy and safety of HIFU treatment [6,7,8]. In the published literature, there are discrepancies in such adverse effects observed in clinics. No major injury in the inferior vena cava, main hepatic vein branches, and the portal vein and its main branches was found in 39 patients with 42 hepatocellular carcinomas in the size range of 1.5–22 cm after 23.8 ± 17.2-month follow-up of HIFU ablation, with the distance between the tumor and main blood vessel being less than 1 cm [9]. However, after HIFU treatment of a group of 164 patients with malignant solid tumors, an abrupt blood interruption and, subsequently, the cessation of blood flow within the tumor vessel occurred, and both coagulative necrosis and severe damages were found in small tumor vessels [10]. Such changes in vascular integrity and blood flow cause neoplastic tissue ischemia and may play a critical role in secondary tumor cell death due to the destructive capability of HIFU on solid tumors. Another strategy is the occlusion of relatively large vessels supplying tumors, either by itself or in conjunction with anti-cancer agents for the tumor neovasculature. In a meta-analysis of HIFU ablation, there was 13% to 80% occurrence of vessel rupture at the acoustic intensity range of 1690 W/cm^2^ to 8800 W/cm^2^ and at the frequency of 1.49 MHz to 2.2 MHz, which increased with the acoustic intensity [11]. However, tumor hemorrhage or rupture of a large artery was never detected. Hemorrhage caused by rupture of capillaries after HIFU exposure to the brain [12,13] and small vessel (up to 0.2 mm in diameter) in the liver and implanted tumors for occlusion [14] was also observed. Vessel injury occurs only at ultrasonic exposures above the acoustic cavitation threshold, and, below this threshold, only small vessels are damaged.

In our previous study, silicon tubing (inner diameters of 0.76 mm and 3 mm) at varied flow speeds (17–339 cm/s) was used as a vessel phantom [15]. Lesion formation around tubing embedded in the polyacrylamide gel phantom by HIFU ablation was photographically recorded using a high-speed camera. Bubble cavitation and the formation of a liquid jet may be one of the major mechanisms of tubing rupture. To further confirm such observations in more closely clinical conditions, *ex vivo* porcine femoral artery samples were used in this study. Lesion formation with HIFU focus aligned to the anterior wall, middle of the artery, and the posterior wall of the artery was recorded and then quantitatively compared with that without the embedded artery. In addition, high-speed photography was applied to observe the lesion formation and cavitation activities (bubble formation, expansion, motion, and shrinkage) at different testing conditions.

## 2. Materials and Methods

### 2.1. Artery Extraction

The femoral arteries were dissected from fresh porcine leg bought from a local grocery store. Cautious dissections were carried out using a surgical knife, scissor, and tweezers in the lab at room temperature. Initially, the skin and muscle were cut till the surface of the artery could be visualized. Then, the surrounding fats and connective tissue were carefully trimmed away from the artery using surgical scissors. Due to the complex anatomy of the porcine leg and delicate artery structure with the wrapping connective tissue and fats, a long time period (i.e., a few hours for each leg) may be required for this process. The outer diameter of the obtained porcine artery was measured to be 2.95 ± 0.76 mm using a digital caliper (500-196-30, Mitutoyo, Aurora, IL, USA), which is classified as a medium artery [16]. The intactness of the artery was checked by connecting it with a polyvinyl chloride (PVC) rubber tube and injecting the water through it with a 20 mL syringe (BD Biosciences, Singapore). If leaking from the artery wall was found, the sample was not used in the following experiment. If the extracted artery was not used immediately, the sample had to be stored in an airtight bag and placed in a freezer (H275X, Fisher & Paykel, Singapore) at −20 °C immediately to prevent any further moisture loss and sample degradation for future use.

### 2.2. Gel Phantom and Vessel Model Preparation

The optically transparent gel phantom was composed of polyacrylamide hydrogel and bovine serum albumin (BSA) and became optically opaque when thermally denatured at a temperature over 65 °C [17]. Firstly, ultra-pure-grade Tris(hydroxymethyl)aminomethane base (Sigma-Aldrich, Singapore) was added to distilled water and mixed using a magnetic stirrer (Hei-Standard, Heidolph Instruments GmbH, Schwabach, Germany) for around five to ten minutes, allowing full dissolution. The pH value of Tris solution (Sigma-Aldrich) was measured by a research-grade pH meter (Accumet Research AR20, Fisher Scientific, Singapore) based on the three pH buffers of 4, 7, and 10. The pH value of the Tris buffer was adjusted by adding 37% hydrochloric acid (HCI) solution (Sigma-Aldrich). Then, 40% *w*/*v* acrylamide solution (ICN Biomedicals, Aurora, OH, USA) was mixed with 10% *w/v* ammonium persulfate solution (Sigma-Aldrich, Munich, Germany) and 7% BSA (Axil Scientific, Singapore) by weight, degassed for around 1 h in a desiccant chamber (420100000, Scienceware, Pequannock, NJ, USA) with a vacuum pump (VTE8, Thomas, Sheboygan, WI, USA) at a pressure of 150 mbars for degassing the mixture, and then poured into a mold with an acoustically transparent membrane at the bottom for polymerization at room temperature within 1 min of adding N,N,N′,N′-tetra-methylethylene/diamine (TEMED, Sigma-Aldrich, Munich, Germany). A short period of around 10 min to 15 min was sometimes needed for the phantom to solidify. Protective gloves and clothing had to be worn during the fabrication process because acrylamide is a neurotoxin.

**Figure 1:**
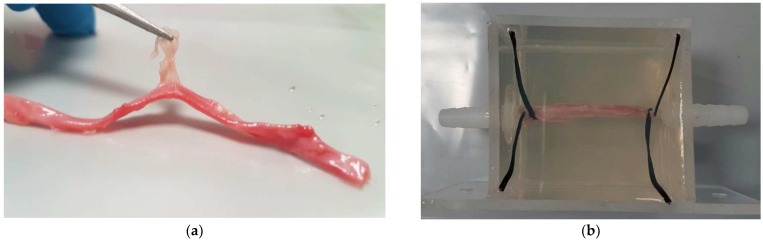
Illustration of an artery (**a**) extracted from the porcine leg and (**b**) connected with fluid connector and embedded in the polyacrylamide gel phantom.

Clear acrylic plastic sheets with a thickness of 3 mm were used as the building material and glued to the phantom model with dimensions of around 60 mm × 40 mm × 50 mm using an all-purpose epoxy (Pioneer, Singapore). Two straight tube connectors (Cole-Parmer, Vernon Hills, IL, USA) were fixed onto the mold using the epoxy, and the artery was connected to it and routed between the connector barbs (sometimes, binding wires were used to enhance the attachment). This epoxy provided better holding strength between acrylic sheets and between the tube connector and clear acrylic sheet. The porcine artery was connected with the tube connectors inside the model first, smeared with ethanol (Sigma-Aldrich, Munich, Germany) on the outer surface to decrease the surface tension and minimize the bubble adhering to the artery wall, and then the BSA gel mixture was poured into the model. After the solidification of polyacrylamide hydrogel, the artery was embedded inside the gel phantom (see Figure 1). Tube connectors on the outside of the model were connected to large silicone tubing (EW-96101-18, Cole-Parmer, Vernon Hills, IL, USA), which was driven by a peristaltic pump (pump head: YZ1515x, BT300-2J, Longer Precision Pump, Baoding, China). Degassed and deionized water (*O_2_* < 4 mg/L, *T* = 25 °C, measured by DO700, Extech Instrument, Waltham, MA, USA) flowed from the reservoir to a fluid collector through the artery sample at a flow rate of 20 mL/min to mimic blood flow.

### 2.3. Experimental Setup

**Figure 2:**
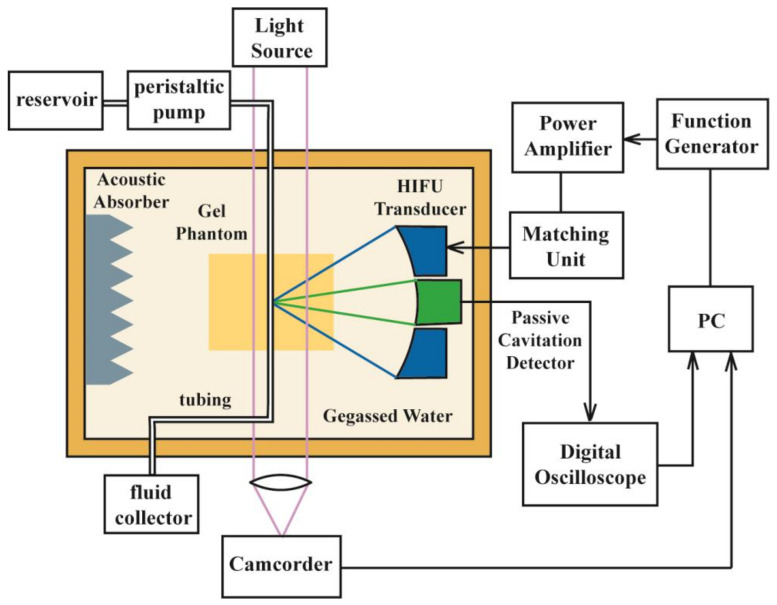
Schematic diagram of HIFU ablation experimental setup with the polyacrylamide gel phantom.

A single-element concave HIFU transducer (H-102, outer diameter = 69.94 mm, inner diameter = 22.0 mm, *F* = 62.64 mm, *f* =3.3 MHz, Sonic Concepts, Woodinville, WA, USA) working at its third harmonic frequency (3.3 MHz) was attached to a three-axis positioning system (PT3/M, Thorlabs, Newton, NJ, USA) for alignment and immersed in the degassed and deionized water of a Lucite tank (L × W × H = 70 cm × 50 cm × 30 cm). Sinusoidal bursts produced by a function generator (AF3021B, Tektronix, Beaverton, OR, USA) together with a power amplifier (BT00250-AlphaA, Tomco Technologies, Adelaide, Australia) and an impedance matching network were used to drive the transducer. In order to minimize the effect of the acoustic reflection, an acoustic absorber was placed on the opposite wall of the testing tank (see Figure 2). The HIFU focus was first aligned to the anterior wall of the artery sample using the pulse-echo method and then moved to the middle and posterior wall of the artery by moving the translational stage axially forward by the radius and diameter of the sample, respectively. According to the acoustic reflecting properties of the porcine artery (two echoes from the anterior and posterior wall, respectively), the radius and diameter of the porcine artery embedded in the optical transparent phantom gel could be determined using the flight-of-time principle and the consistent speed of sound (i.e., 1485 m/s). Bubbles, if existing inside the tubing and artery sample, could be removed by the flushing fluid, and the HIFU focus was shifted transversely by at least 7 mm to minimize the influence of the previous ablation. Because of the uneven surface of the artery sample, alignment was checked before each HIFU ablation. A LabView program (National Instruments, Austin, TX, USA) was applied to set the HIFU parameters (pulse duration of 32 ms, the pulse repetition frequency of 5 Hz, and total exposure of 100 s) and control the exposure.

### 2.4. Photography

A digital camera (Coolpix P510, Nikon, Tokyo, Japan) was used to capture a high-resolution image of the porcine artery and optical opaque lesion immediately after HIFU ablation. A ruler was attached to the wall of the phantom holder for calibration purposes. All captured images were processed in digital image processing software (ImageJ, National Institute of Health, MD, USA) to quantitatively determine the lesion size. Close-up observation of the porcine artery surface was done under an inverted optical microscope (SZX12, Olympus, Tokyo, Japan). Abnormal contour or damage to the surface created by HIFU ablation was found as the microspore and saved as an image for further investigation. Accurate measurement was performed using the calibration of the magnification lens and the measurement scale setting in the Olympus viewer program.

The water tank had two viewing glass windows with a diameter of 90 mm for photography during the experiment. The lesion formation in the BSA gel phantom, the rupture of the artery, the dynamics of the induced bubble, and its interaction with the artery wall were recorded using a high-speed camera (Fastcam 1024PCI, Photron, San Diego, CA, USA) with a magnification lens (Optem Zoom 70Xl 7:1 micro-inspection zoom lens system, Qioptiq Photonics GmbH, Munich, Germany) at a resolution of 1024 by 1024 pixels at a frame rate of 600 Hz. A 2000 W light (LX601GS, Unomat GmbH, Reutlingen, Germany) was used as the illumination during the photography. A transistor–transistor logic (TTL) pulse from a digital delay generator (DG535, Stanford Research Systems, Sunnyvale, CA, USA) was used to synchronize the photography with HIFU ablation. Each frame could be extracted from the captured videos using software (FASTCAM viewer program, Photron) for analysis. At most, two seconds of capturing were available because of the limited memory in the high-speed camera.

### 2.5. Statistical Analysis

The statistical difference between the groups was determined using an analysis of variance (ANOVA) test in SigmaPlot (Systat Software, San Jose, CA, USA), and the significance was fixed at *p* < 0.05. At least five samples were used at each testing condition.

## 3. Results

### 3.1. HIFU-Induced Lesion

**Figure 3:**
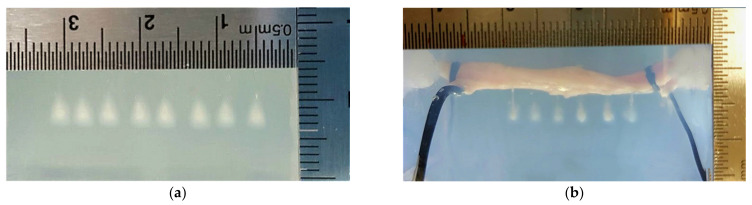

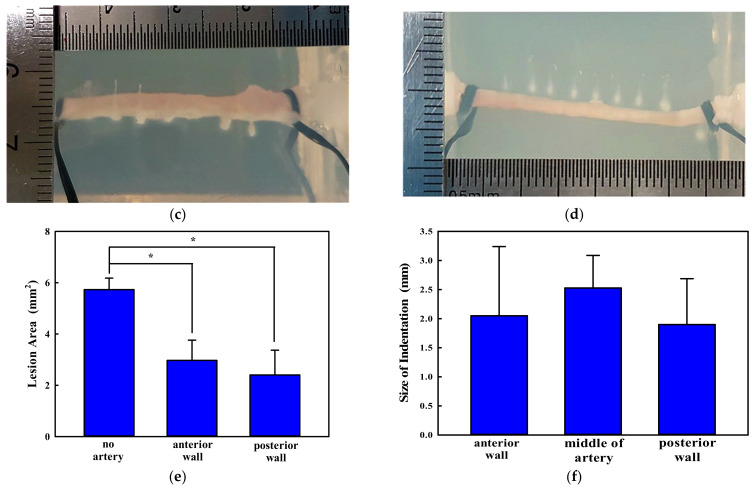
Representative images of HIFU-induced lesion: (**a**) without the inclusion of an artery in the gel phantom, compared to the HIFU focus aligned to (**b**) the anterior artery wall, (**c**) middle of the artery, and (**d**) the posterior artery wall. Comparison of (**e**) HIFU-induced lesion area in the polyacrylamide gel phantom without the inclusion of the artery, HIFU focus aligned to the anterior and posterior artery wall, and (**f**) the size of indentation on the artery wall with the HIFU focus aligned to the anterior wall, middle of the artery, and the posterior wall, *: *p* > 0.05.

The peak positive and negative pressures of the HIFU transducer at the focus in the degassed water are around 34.2 and −11.9 MPa, respectively [12], with a −6 dB beam size of 4 mm × 0.5 mm (axial × lateral) as determined by scanning a needle hydrophone (HNA-0400, Onda, Sunnyvale, CA, USA), following the established measurement protocol [18]. The gel phantom absorbed the acoustic energy at the HIFU focus so that the denaturalization of the BSA protein starting at around 60–65 °C as the opaque lesion was in the shape of a symmetric cigar. Then, the generation of small cavitation bubbles by HIFU ablation scattered the consequent HIFU pulses towards the transducer and accumulated acoustic energy and heat away from the focal point. As a result, the lesion shape changed to resemble a tadpole, with its head continuously growing with the progression of HIFU ablation. HIFU-induced lesions in the gel phantom without porcine artery had a similar size, shape, and position (see Figure 3a). The size of tadpole lesions was around 4.37 ± 0.16 mm in length and 2.70 ± 0.16 mm in width, and the lesion area was 5.73 ± 0.45 mm^2^.

With the inclusion of the porcine artery in the gel phantom, the generated HIFU lesions were significantly different (see Figure 3). When the HIFU focus was aligned to the posterior artery wall, the lesion size decreased to 2.90 ± 0.64 mm (by 34% reduction) in the axial direction and to 1.58 ± 0.49 mm (by 42% reduction) in the lateral direction with a lesion area of 2.97 ± 0.79 mm^2^ (by 48% reduction, *p* < 0.05). If the HIFU focus was aligned to the middle of the artery, significant changes to the shape were found. There were sharp and small tadpole tails stemming from the posterior artery wall and a large tadpole head attached to the anterior artery wall. However, around 70% (14 out of 20) of such tails were not found. Because no lesion could be generated in the circulating fluid, the lesion area was not measured under this testing condition. The diameter of the tadpole head was reduced by 32% to 1.84 ± 0.53 mm. When the posterior artery wall was focused for HIFU ablation, the opaque lesion showed a size of 3.25 ± 0.48 mm in the axial direction (by 26% reduction) and 1.42 ± 0.42 mm in the lateral direction (by 48% reduction) with a lesion area of 2.40 ± 0.97 mm^2^ (by 58% reduction, *p* < 0.05 in Figure 3e,f). In comparison to the other testing conditions, more tiny bubbles were pushed to the tadpole tail according to the acoustic radiation force (illustrated in more detail later). The large variations in the generated lesions may be due to an error in HIFU focus alignment, the varied thickness of the artery wall, and uneven artery surface.

### 3.2. Morphological Changes in the Porcine Artery

**Figure 4:**
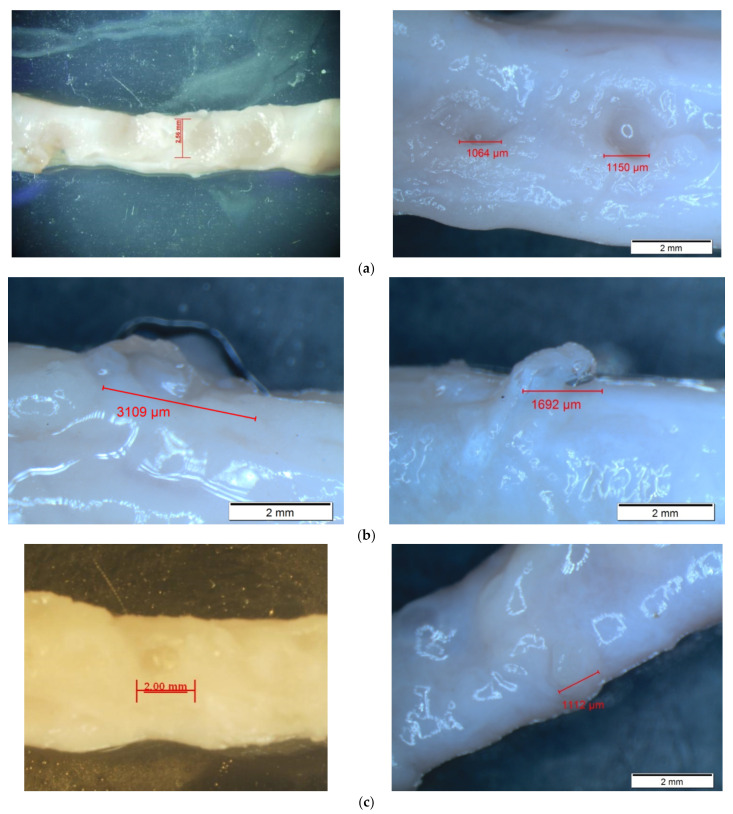
Representative images of HIFU-induced morphological changes to the artery wall with the HIFU focus aligned to (**a**) the anterior artery wall, (**b**) the middle of the artery, and (**c**) the posterior artery wall.

After HIFU ablation, the hydrogel materials were removed from the phantom holder, and the porcine artery samples were released from the fluid connectors. Then, the morphological changes on the artery’s surface were observed under the inverted microscope and distinguishable in comparison to that of the freshly extracted one. There were two types of distinctive differences on the artery surface: indentation and discoloration. At the position of HIFU focus, small pockets of indentation on the tunica adventitia layer (the outermost layer of the artery), which may have been due to bubble cavitation and mechanical damage, were found, with discoloration of the artery, changing from a pinkish involuntary muscle fiber to brown as a sign of thermal damage. The discoloration region was exactly within the indentation area. However, there were no significant signs of deep tissue damage or tear on the surface (see Figure 4). The sizes of HIFU-induced indentation on the porcine artery under these three testing conditions are similar (2.05 ± 1.19 mm vs. 2.53 ± 0.57 mm vs. 1.90 ± 0.79 mm). Although the indentation induced by HIFU ablation with the focus aligned to the middle of the artery seemed larger than the others, there was no statistical difference (*p* > 0.05 in Figure 3f), which may be due to large variations in the experimental data. Among all morphological changes, those induced by HIFU ablation with the focus aligned to the anterior wall had the least discoloration (light brown), while those aligned to the middle of the artery were the most discolored (dark brown). No leakage or punctures were found in any of the treated artery samples.

### 3.3. High-Speed Images

**Figure 5:**
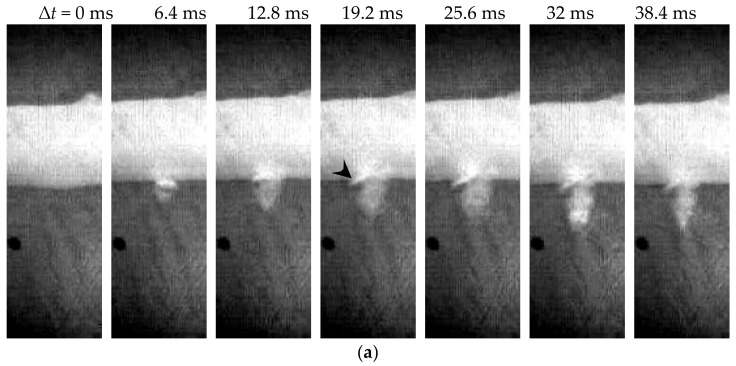

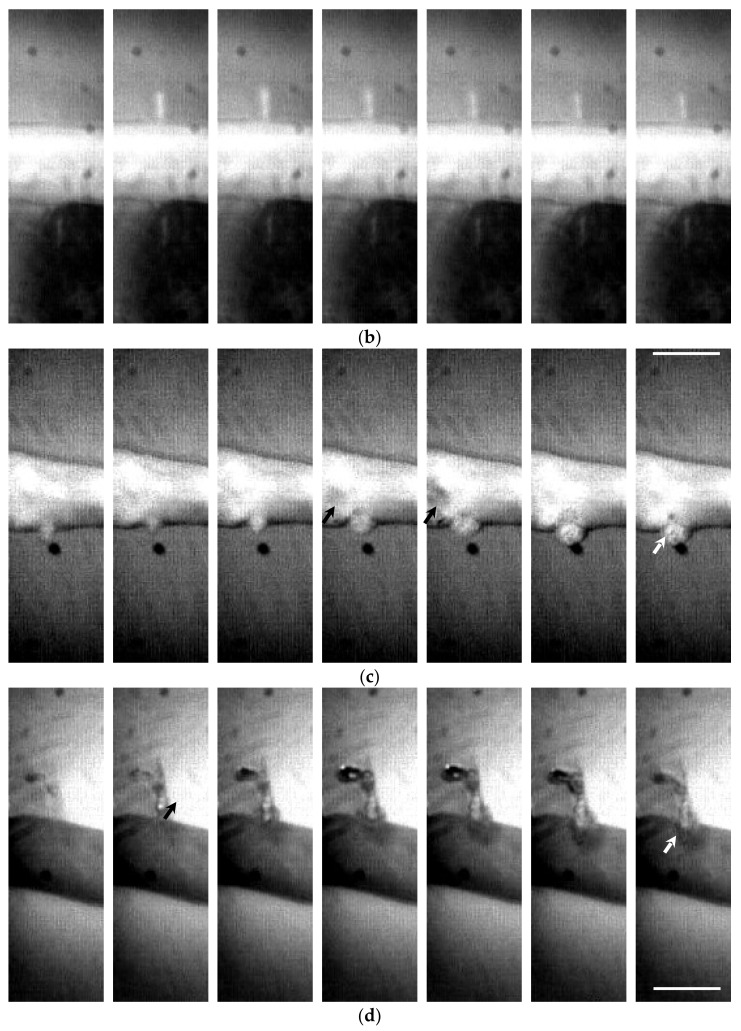
Representative high-speed images of HIFU exposure with its focus aligned to (**a**) the anterior artery wall by the 1st pulse, the middle of the artery by (**b**) the 4th pulse and (**c**) the 3rd pulse (different artery sample here), (**d**) the posterior wall by the 4th pulse. The arrowhead shows the significant interaction with the artery wall, the black arrow shows the motion of the HIFU-induced bubbles to the circulating fluid inside the artery sample, while the white arrow shows the lesion produced by the hydrodynamic cavitation. Scale bar represents 2 mm.

High-speed imaging was only done in the first 11 pulses of HIFU ablation due to the limited memory of the employed camera. The lesion generation due to the increased opacity of the BSA polyacrylamide gel phantom and cavitation activities, such as the bubble generation, expansion, and motion, as well as its interaction with the artery wall, was closely monitored. The ablative lesion growth with the HIFU focus aligned to the artery was significantly different from that in the gel phantom without an embedded artery (see Figure 5). When the HIFU focus was aligned to the anterior surface of the artery wall, the lesion was produced in the first frame (1.6 ms, data not included) after the HIFU pulse arriving the focus. Then, a significant interaction of the HIFU pulse and bubbles with the artery wall was observed, as shown by the arrowhead. The lesion grew towards the transducer and its opacity increased continuously with the progression of HIFU ablation. After the termination of HIFU ablation at ∆*t* = 32 ms, the lesion seemed to shrink slightly but did not disappear (i.e., ∆*t* = 38.4 ms in Figure 5a). The induced bubbles took a long time (ten to a hundred ms) for complete dissolution. If the HIFU focus was aligned to the middle of the artery, two types of lesions were found: a tiny and slim lesion through the posterior wall (Figure 5b) and a large semi-circular one at the anterior wall (Figure 5c). Meanwhile, a large cloud of bubbles was observed to be induced in the circulating fluid inside the artery, as shown by the black arrow in Figure 5b. After HIFU ablation, bubbles were retracted towards the transducer and caused lesion growth again due to the hydrodynamic cavitation (white arrow in Figure 5c) [19]. When the HIFU focus was aligned to the posterior artery wall, bubbles could be produced more easily by HIFU ablation and then pushed forward by the acoustic radiation force. However, they did not always move in the axial or wave-propagating direction of the HIFU transducer as shown by the black arrow in Figure 5d, which may be due to the inhomogeneity of the gel phantom and pre-existing lesions generated by the previous pulses. The hydrodynamic cavitation produced significant distortion and interaction with the posterior artery wall (white dashed arrow in Figure 5d).

**Figure 6:**
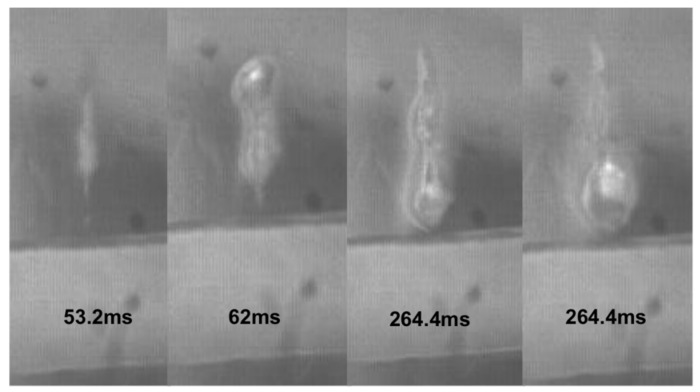
Representative high-speed images of HIFU exposure with its focus aligned to the posterior artery wall.

**Figure 7:**
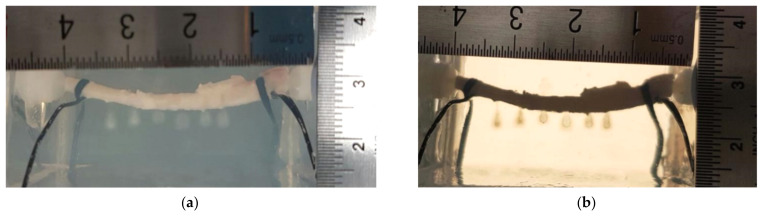
Representative images of HIFU-induced lesions with the HIFU focus aligned to the anterior artery wall using the (**a**) reflecting light and (**b**) transmitting light.

A large boiling bubble was produced in the gel phantom in the current investigation (see Figure 6). Initially, the HIFU-induced bubble was pushed forward by the acoustic radiation force. At the end of HIFU ablation, it retracted towards the transducer. Because complete bubble dissolution depends on the bubble size, such a large bubble remained in the lesion; when the next pulse was delivered, the acoustic energy was scattered/reflected. As a result, the residual bubble grew gradually. These characteristics are similar to the observations made in the gel phantom without an embedded artery [19]. After HIFU ablation, the lesion had a clear dark boundary inside, as shown using the transmitting light (see Figure 7). Inside this boundary is mechanical emulsification, while, outside, thermal coagulation occurs [19].

## 4. Discussion

The establishment of the safety limits of a therapeutic modality is a critical issue for clinical application. To avoid the potential adverse effects of HIFU ablation, one of the requirements specified by the FDA is to keep a safe margin to the major artery (i.e., 1 cm). However, the interaction between HIFU insonation and vascular structure in the tumor always exists. Vascular contraction without tissue degeneration, histological tissue degeneration, and occlusion of the blood flow may occur at low, moderate, and high intensity of ultrasound, respectively. Although these phenomena appeared to be mainly due to thermal effects, mechanical effects also play some roles, particularly in vascular contraction [20]. Hemorrhage within the central nervous system during the therapeutic opening of the blood–brain barrier (BBB) can lead to catastrophic neurological damage. Influences of the artery on HIFU ablation are critical concerns in clinical applications. Although the lesion size reduction in the axial reduction in our previous study with the HIFU focus aligned to the anterior silicone tubing wall is similar to that in the *ex vivo* study (30% vs. 34%), the lateral reduction is much smaller (6% vs. 42%) [15]. Furthermore, the HIFU focus was aligned to the middle and the posterior wall of the artery. The lesion reduction in the axial and lateral direction with the HIFU focus aligned to the posterior artery wall is 26% and 48%, respectively. In contrast, a semi-circular lesion head was usually found on the anterior artery wall and a tiny lesion tail seldom presented on the posterior wall when the HIFU focus was aligned to the middle of the artery, which is mostly because of the thermal diffusion of the circulating fluid inside the artery. In addition, lesion production and bubble dynamics were captured using high-speed photography. The motion of the bubbles by acoustic radiation force and hydrodynamic cavitation, generation of the boiling bubble, interaction with the artery wall, and leakage of bubbles into the circulating fluid were observed. Boiling histotripsy results in tissue homogenization at the center and a thermal lesion at the boundary. These findings may illustrate the influence of vascular structure on HIFU-induced tumor ablation, although no rupture of *ex vivo* artery samples was found in this study.

Cavitation is a common phenomenon in acoustical application. HIFU-induced bubbles are classified as acoustic and boiling ones. When the tensile pressure is larger than the intrinsic threshold, bubbles will be generated and then grow gradually during the tensile cycle and by rectified diffusion through the bubble wall [21]. Acoustic cavitation-induced bubbles enhance the heating effect of sonication by mechanisms such as acoustic scattering/reflection and thermal and viscous damping [22]. However, these bubbles are quite small, in the size of up to a few hundred μm. Acoustic cavitation can also be categorized as stable and inertial (transient) cavitation according to its dynamic characteristics. Stable cavitation or the sustained large-amplitude oscillation of microbubbles produces significant microstreaming [23,24]. Inertial cavitation or the violent collapse of microbubbles causes high-velocity microjets and localized high pressure and temperature, causing substantial mechanical damage of the tissue [23]. Temperature elevation of the tissue/hydrogel target to around 100 °C leads to boiling and a change in the state of water from liquid to vapor. In contrast, boiling bubbles are much larger. The interaction of such boiling bubbles with the subsequent HIFU pulses results in the erosion of soft tissue into the subcelluar size, which is termed boiling histotripsy [25]. Boiling can also cause mechanical damage such as cracking in thermally ablated tissue [26]. Even at short duration, radiation force, streaming, and mechanical effects of cavitation play a concomitant role in mechanical tissue damage [27]. In comparison, hydrodynamic cavitation occurs by the cessation of HIFU exposure. The retraction of the residual bubble would enlarge the erosion area of boiling histotripsy [19]. Cavitation could be detected by using passive cavitation detection (PCD) or passive cavitation imaging (PCI) [28]. Stable cavitation is signified by sub-harmonic and ultra-harmonic emissions, while inertial cavitation by broadband acoustic emissions is associated with bubble collapse [29]. Tissue vaporization can be detected by low-frequency emissions [30]. It was found that sustained cavitation accompanied by sub-harmonic emissions played a central role in vessel hemorrhage during HIFU application for vessel occlusion and its predictive accuracy was higher than that of broadband and low-frequency emissions [31]. A real-time feedback controller to reduce acoustic cavitation by modulating HIFU intensity has great potential to suppress vessel rupture during HIFU ablation.

One possible mechanism of vessel rupture is supratherapeutic heating of the vessel wall. Temperatures around 54 °C caused vessel wall contraction (spasm) and mean temperatures of 77 °C for 3–4 s caused tissue welding. It was found that retreatment of HIFU-induced constricted vessels generated hemorrhage, which may be due to less dissipated heat by reduced convection [32]. Temperatures above l00 °C caused severe tissue disorganization and perforation of the vessel wall during laser ablation in rat carotid arteries [33]. HIFU has been proven to have the capability of vessel occlusion. Occlusion may be due to several mechanisms, such as narrowing of the lumen by vascular spasm and acoustic radiation force, coagulation of collagen in the vessel wall, edema of surrounding tissue, thermal and mechanical damage to the endothelium, activation and aggregation of platelets, and formation of intraluminal thrombus. With vessel occlusion and the suppression of thermal diffusion by the circulating blood, the temperature induced by HIFU ablation will become much higher. In addition, the loss of vascular elasticity provided by fibrin and disorganization and delamination of the cells was found in the occluded tumor [34]. In comparison to the *in vivo* environment, the *ex vivo* artery model may not have those responses mentioned above, and no blood was circulated inside the artery here for triggering the occlusion. Therefore, the observation of thermal effects in the *ex vivo* studies cannot be extrapolated to *in vivo* directly.

After HIFU ablation, both mechanical and thermal effects on the treated artery wall were visible under the microscope. Discolorations on the artery were a sign of thermal reaction, while the pocket of indentation was caused by the mechanical effects of HIFU ablation. Evidence of cavitation activities, the interaction between bubble and artery, and motion of the artery wall by the acoustic radiation force is available in the captured high-speed images. Variations in the lesion and cavitation patterns are due to the heterogeneity of *ex vivo* tissue samples and the randomness of the cavitation itself. According to the results of this study, no rupture was found in the treated artery in all testing conditions despite a clear sign of damage on the artery surface. Microscopic observations showed no penetration of the artery, so it is highly possible that only the tunica media and tunica adventitia layer of the artery were affected by HIFU ablation. Leakage of bubbles into the circulating fluid, as shown in Figure 5c, may be an indicator of vessel rupture, similar to that found in the silicone tubing during HIFU ablation [15]. However, this rupture may be healed by the tissue constriction in the temperature elevation. Further examination, such as histopathological evaluation and electron microscopic observation, is required to confirm this hypothesis and investigate the structural changes to the treated tissue, which is difficult under light microscopy.

**Figure 8:**
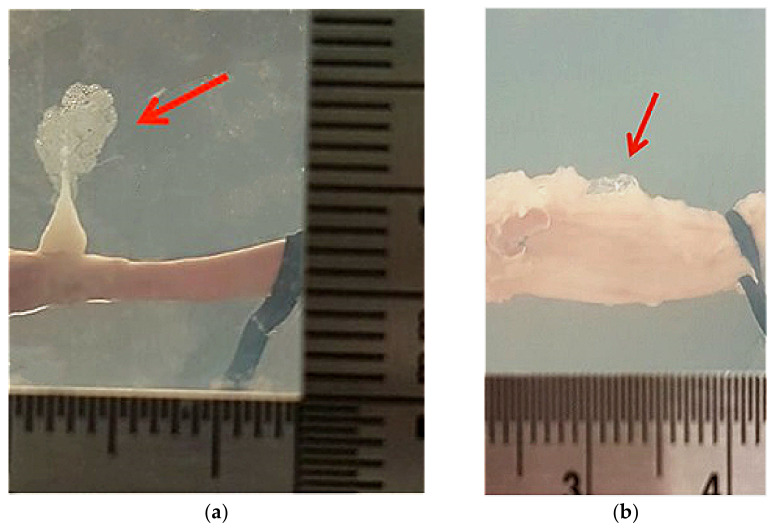
Abnormal phenomena of (**a**) lesion in a shape of a “squid” with a slim structure in the lesion tail and (**b**) large residual bubble on the artery without lesion surrounding it found after HIFU ablation.

Two abnormal results were also found after HIFU ablation, but the two porcine arteries used in the experiment had no significance over the others, a similar uneven surface contour, diameter, and slight movement during the insonation. A slim, flat piece of mesentery-like object appearing at the sharp lesion tail in the shape of a “squid” (see Figure 8a) [19] and a very large bubble remained on the surface of the artery without lesion around it (see Figure 8b). The underlying mechanisms of these are unclear and need further investigation.

## 5. Conclusions

In this study, the influences of the vessels on the lesion production and potential of vessel rupture during high-intensity focused ultrasound (HIFU) ablation for solid tumors and cancers were investigated *ex vivo*. The observations on the silicone tubing in our previous study cannot be extrapolated to the artery sample. The presence of extracted porcine artery in the polyacrylamide gel phantom leads to a decrease in lesion size in both the axial and lateral directions. In comparison to the lesion size reduction for HIFU ablation to silicone tubing, the axial reduction is similar, but the lateral reduction is much larger. The characteristics of the lesion are highly dependent on the focus alignment to the anterior wall, middle of the artery, and posterior wall. From the high-speed photography, the generation, expansion, motion, and shrinkage of bubbles and their interaction with the artery were recorded and compared. It was found that both acoustic and hydrodynamic cavitation are critical in lesion production and have a significant interaction with the artery. After HIFU ablation, both thermal and mechanical effects were found on the surface of the artery wall, but without any rupture. In contrast, rupture on the silicone tubing was found but without significant discoloration on the surface. Altogether, cavitation and boiling bubbles play an important role in the HIFU-induced damage on the artery. Controlling them appropriately in real time may be a good strategy for enhancing the safety and efficacy of HIFU cancer ablation in clinics.
